# The dysregulation of miRNAs in epilepsy and their regulatory role in inflammation and apoptosis

**DOI:** 10.1007/s10142-023-01220-y

**Published:** 2023-08-31

**Authors:** Guoping Xie, Huan Chen, Chan He, Siheng Hu, Xue Xiao, Qunying Luo

**Affiliations:** 1 Department of Clinical Laboratory, The Second Staff Hospital of Wuhan Iron and Steel (Group) Corporation, Wuhan, Hubei China; 2 Department of Clinical Laboratory, Wuhan Institute of Technology Hospital, Wuhan Institute of Technology, Wuhan, China; 3 Department of Clinical Laboratory, Maternal and Child Health Hospital in Wuchang District, Wuhan, Hubei China; 4 Department of Clinical Laboratory, Honggangcheng Street Community Health Service Center, Qingshan District, Wuhan, Hubei China; 5 Department of Clinical Laboratory, Gongrencun Street Community Health Service Center, Wuhan, China; 6 Department of Neurology, Huarun Wuhan Iron and Steel General Hospital, Wuhan, Hubei China

**Keywords:** Epilepsy, miRNA, Inflammation, Apoptosis

## Abstract

Epilepsy is a neurological disorder that impacts millions of people worldwide, and it is characterized by the occurrence of recurrent seizures. The pathogenesis of epilepsy is complex, involving dysregulation of various genes and signaling pathways. MicroRNAs (miRNAs) are a group of small non-coding RNAs that play a vital role in the regulation of gene expression. They have been found to be involved in the pathogenesis of epilepsy, acting as key regulators of neuronal excitability and synaptic plasticity. In recent years, there has been a growing interest in exploring the miRNA regulatory network in epilepsy. This review summarizes the current knowledge of the regulatory miRNAs involved in inflammation and apoptosis in epilepsy and discusses its potential as a new avenue for developing targeted therapies for the treatment of epilepsy.

## Introduction

Despite significant research efforts, the intricate mechanisms underlying epilepsy remain incompletely understood. Epilepsy, a neurological condition characterized by recurring and unpredictable seizures, which is thought to arise from complex dysregulation of genes and signaling pathways (Kobylarek et al. 2019). The development and manifestation of epilepsy involve a diverse array of factors and processes, collectively contributing to the pathogenesis of the disorder (Cheroni et al. 2020). These may include genetic mutations, altered neuronal excitability, imbalances in neurotransmitters, synaptic abnormalities, inflammatory responses, and disrupted network connectivity (Rho and Boison 2022; Specchio et al. 2022; Yang et al. 2018). Seizures are categorized into three groups: generalized, focal (formerly referred to as partial), and epileptic spasms. Focal seizures initiate within specific neuronal networks confined to a portion of one cerebral hemisphere. Generalized seizures commence in widely distributed bilateral neuronal networks. It's possible for a seizure to commence as focal and subsequently become generalized. Seizures can emerge within the cortex or subcortical structures. By utilizing comprehensive medical history, EEG results, and supplementary data, a physician can frequently classify the type of seizure or epilepsy. This paves the way for a suitable diagnostic assessment and the creation of an appropriate treatment strategy (Stafstrom and Carmant 2015). The most recent categorization of seizures and epilepsies was introduced by the International League Against Epilepsy (ILAE) in March 2017. This updated classification offers improved organization, providing clear definitions of terminology and introducing novel seizure types. Effective diagnosis and management of seizures and epilepsy benefit from this structured grouping, as distinct medications tend to target specific seizure types (Dhinakaran and Mishra 2019). Under the ILAE's current classification, the clinical aspects of epilepsy are organized into three tiers: the individual seizures, the overarching epilepsies, and the comprehensive epilepsy syndromes. This approach takes into account the underlying causes and associated conditions at each level (Zuberi and Brunklaus 2018). Furthermore, a noteworthy shift labels epilepsy as a treatable condition rather than a disorder. Resolution is determined after a ten-year period of being seizure-free, including the last five years without medications, or when the patient is no longer susceptible to age-related epilepsy syndromes (Fisher and Bonner 2018). However, the precise interplay between these factors and their specific contributions to epileptogenesis and seizure generation are still being actively investigated. Ongoing research efforts strive to unravel the intricate nature of epilepsy and enhance our comprehension of the molecular and cellular mechanisms that underlie the disorder, which ultimately leading to improved diagnostic and therapeutic strategies for individuals affected by this condition.

MicroRNAs (miRNAs) are a group of small non-coding RNAs that have been demonstrated to have a crucial role in the regulation of genes and post-transcriptional control of gene expression across various cellular pathways and systems. (Taft et al. 2010). In 1993, Victor Ambros and Gary Ruvkun made the groundbreaking discovery of the first identified miRNA, lin-4 (Lee et al. 1993). Initially, lin-4 was discovered to have a regulatory role in controlling the timing of larval development in the model organism C. elegans. It was intriguing because it did not encode a protein but instead acted as a small RNA molecule that could control gene expression (Lee et al. 1993). This discovery challenged the long-held notion that RNA molecules solely served as templates for protein synthesis. Subsequently, it was determined that the lin-4 miRNA specifically targets the 3' untranslated region (UTR) of its respective messenger RNA (mRNA) targets. the lin-14 gene. Through complementary base pairing, lin-4 bound to the lin-14 mRNA and inhibited its translation, leading to delayed development in C. elegans (Lee et al. 1993). This landmark discovery paved the way for further exploration of miRNAs as potent regulators of gene expression. Following the discovery of lin-4, numerous miRNAs have been identified in diverse organisms, including humans, amounting to thousands in total. MiRNAs have emerged as key players involved in a broad spectrum of biological processes, encompassing pivotal roles in development, differentiation, proliferation, and apoptosis (Annese et al. 2020). They exert their regulatory effects by binding to target mRNAs, usually at the 3' UTR, leading to mRNA degradation or translational repression (Annese et al. 2020).

The study of miRNAs has provided insights into the complexity of gene regulation and the intricate networks that control cellular processes. Understanding the functions and mechanisms of miRNAs has tremendous implications for human health, as dysregulation of miRNAs has been associated with numerous diseases, including cancer (Saral et al. 2022; Xu et al. 2022), cardiovascular disorders (Hua et al. 2021), neurodegenerative diseases(Nguyen et al. 2022; Ryu et al. 2023), and metabolic disorders (Meerson et al. 2013). Harnessing the therapeutic potential of miRNAs is an active area of research, offering new avenues for developing innovative treatments and diagnostic tools. Recently, there has been a growing focus on the role of miRNAs in the context of epilepsy, as studies have identified several miRNAs that are involved in the pathogenesis of epilepsy, acting as key regulators of neuronal excitability and synaptic plasticity (Feng et al. 2020; Tan et al. 2013; W. Wang et al. 2018a, b). A rising body of research has delved into the regulatory network between miRNAs and mRNAs in epilepsy, offering fresh perspectives into the pathogenesis of the condition and aiding in the advancement of targeted therapies for its treatment.

Understanding the precise origin of epilepsy remains a subject of debate. The progression of this condition seems to be intricately connected to various factors such as neuronal cell apoptosis, the reconfiguration of pathological circuits, proliferation of glial fibroblasts, and inflammatory responses (Chang and Lowenstein 2003; Vezzani et al. 2011). miRNAs are emerging as potential contributors to epilepsy's onset and development by impacting processes such as inflammation, apoptosis, glial cell dysfunction, circuit re-formation, autophagy, oxidative stress, and neurotrophic factor deregulation (Wang and Zhao 2021). By modulating these pathological processes, miRNAs could play a crucial role in the pathogenesis of epilepsy (Cattani et al. 2016). The intricate interplay between these miRNAs and the underlying molecular mechanisms of epilepsy suggests their involvement in orchestrating the complex cascade of events leading to epileptic manifestations.

Brain injury triggers inflammation in the central nervous system and disrupts normal neuronal connections in the hippocampus. Inflammatory disorders affecting the entire body can also cause inflammation in the peripheral system, contributing to the accumulation of inflammatory molecules. This dual inflammation, both peripheral and central, leads to the weakening of the blood–brain barrier due to heightened levels of inflammatory molecules. As a result of the compromised barrier, immune cells infiltrate the brain, promoting increased neuronal excitability and further elevation of inflammatory molecules. When peripheral and central inflammation remain unregulated and the blood–brain barrier is compromised, structural changes occur in the synapses of the hippocampus, ultimately culminating in the development of epilepsy (Rana and Musto 2018). Apoptosis of neurons and glial cells in the brain is of great importance in the pathogenesis of epilepsy, especially the drug-resistant forms. Recurrent epileptic seizures have the potential to trigger neuronal apoptosis, leading to a reduction in cell number. This reduction can lead to a rearrangement of synapses between neurons, forming atypical synaptic circuits that contribute to the recurrence of epilepsy (Henshall and Simon 2005).

The objective of this review is to provide a comprehensive summary of the present understanding regarding the dysregulation of miRNAs in epilepsy and their regulatory role in inflammation and apoptosis. This includes an overview of dysregulated miRNAs and their functions in relation to inflammation and apoptosis, as well as an exploration of their potential as therapeutic targets for the effective treatment of epilepsy.

## The emergence of miRNA-guided effector systems

The biogenesis of miRNAs is a multifaceted process that takes place within the nucleus and cytoplasm of cells. It commences with the transcription of miRNA genes by either RNA polymerase II or III (Lee et al. 2004), producing primary miRNA transcripts (pri-miRNAs). Typically, pri-miRNAs consist of several hundred nucleotides in length and contain a hairpin structure (Cai et al. 2004). Within the nucleus, the microprocessor complex, consisting of the RNase III enzyme Drosha and its cofactor DGCR8, recognizes and processes the pri-miRNAs (Blaszczyk et al. 2001; Han et al. 2004, 2009; Romero-Cordoba et al. 2014). The microprocessor complex carries out cleavage of the pri-miRNA hairpin, resulting in the release of a shorter hairpin precursor called pre-miRNA (Morlando et al. 2008). Following that, the pre-miRNAs undergo transportation from the nucleus to the cytoplasm facilitated by the exportin-5/Ran-GTP complex (Yi et al. 2003). Within the cytoplasm, the pre-miRNAs undergo additional processing mediated by the enzyme Dicer. Dicer cleaves the pre-miRNA hairpin, resulting in the formation of a miRNA duplex. The miRNA duplex comprises two strands: the guide strand, which represents the mature miRNA, and the passenger strand, also referred to as the miRNA* (Feng et al. 2012; Zeng and Cullen 2004). After then, the miRNA duplex is incorporated into an RNA-induced silencing complex (RISC), consisting of Argonaute proteins and other associated proteins. Inside the RISC, the passenger strand is typically degraded, while the guide strand remains stably bound to the Argonaute protein. The mature miRNA within the RISC serves as a guide to target specific messenger RNAs (mRNAs) through complementary base pairing. The binding of the miRNA to its target mRNA can lead to translational repression or degradation of the mRNA, thereby regulating gene expression (Behm-Ansmant et al. 2006; Chendrimada et al. 2005; Cougot et al. 2004; Eulalio et al. 2008; Khvorova et al. 2003; Lee et al. 2006; Liu et al. 2005; Maniataki and Mourelatos 2005; Sheth and Parker 2003).

## Dysregulated expression of miRNAs in the context of epilepsy

In epileptic rats, an experiment demonstrated increased levels of miR-103a and GFAP, elevated apoptosis in neurons, down-regulated BDNF, and reduced numbers of surviving neurons in hippocampal tissues. Suppression of miR-103a resulted in decreased GFAP, increased BDNF, reduced apoptosis, and enhanced neuron survival. Thus, suppressing miR-103a activates astrocytes in the hippocampus and ameliorates neuronal damage in epileptic rats by regulating BDNF expression (Zheng et al. 2019).

Overexpression of miR-27a-3p has been observed in hippocampal cells of epileptic rats and KA-treated neurons. Silencing miR-27a-3p relieved epileptic seizures in animal models, suppressed hippocampal neuron apoptosis, increased Bcl2 expression, and reduced Bax and Caspase3 levels. Additionally, miR-27a-3p silencing effectively decreased IL-1β, IL-6, and TNF-α expressions in hippocampal neurons. These effects were mediated by modulating the expression of MAP2K4, a direct target of miR-27a-3p. Silencing miR-27a-3p also enhanced survival and reduced apoptosis in KA-treated neurons, while influencing MAP2K4 expression. Therefore, miR-27a-3p silencing protects against epilepsy-associated inflammatory responses and hippocampal neuron apoptosis by regulating MAP2K4 expression (Lu et al. 2019).

miR-132 stands out as one of the highly upregulated miRNAs in animal models of temporal lobe epilepsy (TLE) which has the ability to influence the functions of both neurons and glial cells (Korotkov et al. 2020; Yuan et al. 2016). Based on experiments conducted in hippocampal neuron cultures, it has been discovered that the interaction between miR-132 and the p250GAP/Cdc42 axis serves as the underlying mechanism through which this miRNA contributes to epileptogenesis (Yuan et al. 2016). Increased levels of miR-132 have been observed in hippocampal cells of individuals and rat models with epilepsy, with glial cells showing particularly elevated expression. Introducing miR-132 into human primary astrocytes led to reduced expression of several pro-epileptogenic genes, such as COX-2, IL-1β, TGF-β2, CCL2, and MMP3 (Korotkov et al. 2020). In the hippocampal neuron culture model of status epilepticus (SE) induced by Mg (2 +)-deficient medium, miR-132 and BDNF transcripts were significantly elevated. Activation of TrkB.FL with BDNF pretreatment partially suppressed Mg (2 +)-free associated high-frequency epileptiform discharges, while upregulation of miR-132 aggravated such discharges. Additionally, miR-132 contributed to the postepileptic enhancement of high voltage dependent calcium channels. Hence, miR-132 exerts pro-epileptic effects by modulating the BDNF/TrkB pathway in this model (Xiang et al. 2015).

miR-146a, another miRNA that is upregulated in epilepsy, has been observed to display elevated expression in the hippocampal tissues of a rat model of TLE. Knocking down miR-146a significantly improved neuron injury and reduced cell apoptosis in the rat hippocampus. It also decreased levels of MDA, IL-1β, IL-6, and IL-18, while increasing SOD levels in the tissue. Additionally, miR-146a silencing reduced expressions of caspase-9, GFAP, Notch-1, and Hes-1 in the hippocampus of TLE animal models. Functional studies have confirmed Notch-1 as a target of miR-146a. Therefore, silencing miR-146a alleviates neuron injury in the hippocampus of TLE animal models by inhibiting Notch-1 expression (Huang et al. 2019). In the lithium-pilocarpine-induced model of epilepsy, a separate study has shown elevated levels of miR-146a. Suppression of miR-146a has been associated with decreased levels of IL-1β, IL-6, and TNF-α. Additionally, silencing miR-146a has resulted in reduced expressions of P-gp and p-P65/P65, while increasing expressions of Bcl-2/Bax (Deng et al. 2019).

miR-181a, another up-regulated miRNA in epilepsy, has shown to have protective effects when inhibited. Inhibition of miR-181a has led to a reduction in apoptosis, decreased activation of microglia and astrocytes, and upregulation of SIRT1 (Kong et al. 2020). Additionally, silencing of miR-181a has shown to mitigate apoptosis in hippocampal neurons (Ren et al. 2016).

Expression levels of miR-21-5p and mTOR have been found to be elevated in rats across different phases of epilepsy, while PTEN is down-regulated. Inhibiting miR-21-5p in vivo has resulted in the down-regulation of mTOR and up-regulation of PTEN. Furthermore, miR-21-5p suppression has reduced abnormal spikes in EEG, mitigated neuron defects, and improved cognitive and memory impairments associated with epilepsy. The engagement of miR-21-5p in the pathogenesis of epilepsy is linked to its targeting of the PTEN-mTOR axis, which serves as the fundamental molecular mechanism underlying its involvement (Tang et al. 2018).

Notably, around one-third of individuals diagnosed with epilepsy will eventually progress to refractory epilepsy. miR-153 has been found to be associated with the development of refractory epilepsy. Its role potentially involves the regulation of HIF-1α expression, suggesting its contribution to the underlying mechanisms of refractory epilepsy (Li et al. 2016).

miR-34c-5p expression has also been observed to reduce in refractory epilepsy patients, targeting the inflammation-related gene HMGB1. In rat models of kainic acid-induced epilepsy, down-regulation of miR-34c-5p, up-regulation of HMGB1 and IL-1β, and increased hippocampal neuron loss occur in drug-resistant animals. This aggravates neuroinflammation and exacerbates neuron loss, suggesting miR-34c-5p as a potential noninvasive marker for refractory epilepsy. Additionally, dysregulation of miR-153, possibly through HIF-1α regulation, is implicated in refractory epilepsy pathogenesis (Fu et al. 2020).

A decrease in the expression of miR-139-5p has been observed in the serum of children with refractory epilepsy, whereas there is an increased expression of multidrug resistance-associated protein 1 (MRP1). A similar expression pattern has been found in brain samples from rat models of refractory epilepsy. Functional studies have demonstrated that miR-139-5p directly targets MRP1. Transfection experiments in drug-resistant rats' hippocampus have confirmed that up-regulating miR-139-5p or silencing MRP1 reduces neuron apoptosis, enhances neuron survival, and improves neuron injury. These findings suggest that targeting the miR-139-5p and MRP1 axis could potentially overcome drug resistance in refractory epilepsy (Wang et al. 2020).

Li et al. investigated miR-15a-5p expression in serum samples of children with TLE and cultured magnesium-deficient hippocampal cells to simulate TLE. They observed down-regulation of miR-15a-5p in TLE sera and confirmed its suitability as a specific marker for diagnosing TLE in children. The magnesium-deficient condition further reduced miR-15a-5p expression in hippocampal cells, while up-regulation of miR-15a-5p mitigated TLE-induced decreases in cell viability and apoptosis. Thus, miR-15a-5p holds promise as a potential marker for detecting TLE in children (N. Li et al. 2020a, b).

In a rat model of epilepsy, it was found that miR-21-5p binds to STAT3. Inhibition of miR-21-5p led to higher expressions of caspase-3 and Bax, lower expression of Bcl-2, loss of hippocampal neurons, and induction of apoptosis. Conversely, suppressing STAT3 expression produced opposite effects. Additionally, miR-21-5p inhibition increased IL-6 levels. These findings indicate that miR-21-5p is capable of suppressing the expression of STAT3, decrease IL-6 levels, and protect hippocampal neurons from the detrimental effects of epilepsy (Zhang et al. 2020). Table 1 present miRNAs with dysregulated expression in epilepsy.

**Table 1:** Aberrant expressed miRNAs in epilepsy

miRNA	Effects	Study
miR-103a	Increased levels in epileptic rats, triggers GFAP and neuronal apoptosis. Suppression reduces GFAP, increases BDNF, and enhances neuron survival	(Zheng et al. 2019)
miR-27a-3p	Overexpressed in epileptic rats and KA-treated neurons. Silencing relieves seizures, reduces apoptosis, enhances Bcl2, decreases IL-1β, IL-6, TNF-α. Modulates MAP2K4 expression	(Lu et al. 2019)
miR-132	Highly upregulated in animal models of TLE. Targets p250GAP/Cdc42 axis, influences neuron and glial functions. Elevated expression in epilepsy, particularly in glial cells	(Korotkov et al. 2020; Xiang et al. 2015; Yuan et al. 2016)
miR-146a	Elevated expression in rat TLE hippocampal tissues. Silencing improves neuron injury, reduces apoptosis, decreases MDA, IL-1β, IL-6, IL-18, increases SOD levels	(Deng et al. 2019; Huang et al. 2019)
miR-181a	Upregulated in epilepsy, protective effects when inhibited. Reduces apoptosis, microglia, astrocyte activation, upregulates SIRT1. Mitigates hippocampal neuron apoptosis	(Kong et al. 2020; Ren et al. 2016)
miR-21-5p	Elevated in rats during epilepsy phases. Suppression down-regulates mTOR, up-regulates PTEN. Reduces EEG spikes, neuron defects, improves cognition and memory	(Tang et al. 2018)
miR-153	Associated with refractory epilepsy development. May regulate HIF-1α expression, contributing to refractory epilepsy mechanisms	(Li et al. 2016)
miR-34c-5p	Reduced expression in refractory epilepsy. Targets HMGB1. Down-regulation exacerbates neuroinflammation, neuron loss. Potential noninvasive marker for refractory epilepsy	(Fu et al. 2020)
miR-139-5p	Decreased expression in refractory epilepsy patients, targets MRP1. Modulation reduces apoptosis, enhances neuron survival. Overcoming drug resistance potential	(Wang et al. 2020)
miR-15a-5p	Down-regulated in children with TLE. Reduced expression in hippocampal cells. Potential marker for detecting TLE in children	(N. Li et al. 2020a, b)
miR-21-5p	Binds to STAT3, inhibition increases caspase-3, Bax, decreases Bcl-2, triggers apoptosis. Suppresses STAT3 expression, decreases IL-6, protects hippocampal neurons	(Zhang et al. 2020)

## The role of miRNAs in inflammation and apoptosis of epilepsy

miRNAs exert diverse effects on epileptogenesis, with regulation of inflammatory responses and modulation of apoptosis emerging as key mechanisms underlying their involvement in epilepsy pathogenesis.

miRNAs have emerged as vital regulators of innate and adaptive immune responses, and their disrupted regulation within the immune system has been linked to numerous human diseases, including epilepsy. The potential use of miRNAs as biomarkers and innovative therapeutic strategies holds great promise for the future diagnosis and treatment of epileptic disorders. Targeting miRNAs as therapeutic interventions presents several advantages in the field of epilepsy. They can target multiple genes within the same cell, affecting multiple pathways simultaneously. Furthermore, miRNA-mediated processes play pivotal roles in epileptogenesis, encompassing neuronal death, inflammation, gliosis and neuronal microstructure.

In recent years, substantial progress has been made in the field of RNA-based therapeutics, with numerous innovative medicines entering clinical trials. This progress further underscores the potential of miRNAs as therapeutic agents. Additionally, miRNAs possess immense potential as non-invasive biomarkers for diagnosing diseases, predicting prognosis, monitoring treatment response, and stratifying patients. Their stability and ease of detection in diverse tissues, particularly in blood samples, render them highly attractive for clinical applications.

Understanding the complex roles of miRNAs in immune regulation and their involvement in epilepsy not only provides insights into disease mechanisms but also opens up new avenues for targeted interventions. Harnessing the power of miRNAs as diagnostic tools and therapeutic agents may revolutionize the management of epileptic disorders, offering personalized treatment approaches and improved patient outcomes.

miRNAs exert a notable influence on inflammatory pathways that are linked to epilepsy (Hsu et al. 2014; Kan et al. 2012). miRNAs serve as critical regulators of the innate immune response, particularly in modulating inflammation mediated by astrocytes. Harnessing the potential of these miRNAs as innovative therapeutic targets shows promise for the treatment of epilepsy. Additionally, miRNAs are regulated not only in the brain but also in the bloodstream, indicating their potential as biomarkers for brain injury and other neuronal disorders. Blood-based miRNAs offer a non-invasive approach to assess disease status and response to treatment, thereby facilitating improved diagnosis and personalized management of epilepsy (Liu et al. 2010). Asirvatham et al. employed computational approaches to identify miRNA targets in 613 immune genes. Their analysis unveiled around 275 interactions between immune genes and miRNAs, encompassing transcription factors, chromatin modifiers, as well as genes implicated in immune signaling pathways and inflammation, such as cytokines (Asirvatham et al. 2009). Notably, miRNAs were found to exert substantial regulation over the TGF-β pathway, particularly targeting downstream signaling components such as SMAD genes and the corepressor TGIF. Within the TGF-β pathway, 17 out of 24 components were discovered to possess binding sites for 64 distinct miRNAs (Asirvatham et al. 2008). In epilepsy, extensive alterations in miRNA expression have been observed, with a particular emphasis on astrocytes and the immune response as primary targets of deregulated miRNAs. Notably, deregulated miRNAs in epilepsy exhibit a strong affinity for targeting inflammatory mediators, suggesting an involvement of inflammation in the disease. Furthermore, the immune response, along with several other cellular processes implicated in epilepsy, is significantly impacted by deregulated miRNAs, leading to a simultaneous decrease in miRNAs that target the untranslated regions of genes associated with inflammatory mediators (Hu et al. 2011; Kan et al. 2012; Liu et al. 2010; Omran et al. 2012; Wang et al. 2015a, b; Wang et al. 2015a, b).

The involvement of miR-146a in epileptogenesis is associated with its role in regulating the inflammatory response. It functions as part of a feedback system involving NF-κB and MyD88, where NF-κB induction leads to miR-146a upregulation. miR-146a, in turn, downregulates L-1RI-associated protein kinases -1 (IRAK1), IRAK2, and TRAF6, key molecules in the inflammatory pathway downstream of TLR and cytokine receptors (Hou et al. 2009; Taganov et al. 2006). Dysregulation of IRAK1 and miR-146a in opposite directions suggests their involvement in inflammation (Wang et al. 2015a, b). The elevated expression of miR-146a and IL-1β in astrocytes of epilepsy models indicates their involvement in modulating the IL-1β-induced inflammatory response (Aronica et al. 2010; Sheedy and O'Neill 2008; Vezzani et al. 2008). miR-146a is upregulated in patients with mesial temporal lobe epilepsy (mTLE) and plays a role in regulating astrocyte-mediated inflammation. It is also induced in astrocytes by IL-1β stimulation, which is elevated in MTLE animal models. miR-146a functions as a negative-feedback regulator of the inflammatory response by targeting key components such as IRAK-1, IRAK-2, IL-6, TRAF-6, and COX-2. Modulating miR-146a levels affects the expression of these targets and influences the inflammatory response (Aronica et al. 2010; Cui et al. 2010; Hou et al. 2009; Hu et al. 2011; Iyer et al. 2012; Omran et al. 2012; Saba et al. 2014). The findings indicate that miR-146a serves as a negative regulator of TLR signaling, uncovering novel roles for miRNAs in modulating signaling pathways.

miR-155, another miRNA linked to inflammatory pathways in MTLE, shows increased expression in hippocampal tissue of children with MTLE and in an immature rat epilepsy model. Furthermore, the elevated expression of miR-155 coincides with increased levels of TNF-α and ICAM-1 in the nervous tissue (Ashhab et al. 2013).

It has been demonstrated that miR-221 and miR-222 target the 3' UTR of ICAM1, which is instrumental in regulating inflammation and facilitating interactions between immune cells through in vitro experiments (Dietrich 2002). The reduced expression of miR-221 and miR-222 in MTLE + HS is correlated with an increased expression of ICAM1 in astrocytes (Kan et al. 2012). ICAM1 is implicated in various processes, including leukocyte accumulation, microglia recruitment, and cytokine production. Elevated ICAM1 expression has the potential to trigger the release of additional inflammatory mediators and attract immune cells, thereby amplifying the immune response. It has been suggested that posttranscriptional regulation of ICAM1 is involved in MTLE, and the findings of Kan et al. support the idea of miRNA-mediated down-regulation of ICAM1 (Ueda et al. 2009). Additionally, miR-222 has been shown to regulate ICAM1 in glioma cells, highlighting its potential role in modulating ICAM1 expression. Wang's recent studies aimed to identify dysregulated microRNAs associated with inflammation, diagnosis, and drug resistance in serum.

In the context of Alzheimer's disease (AD) and multiple sclerosis (MS), let-7d-5p has been identified as a dysregulated miRNA. Particularly in MS, there is a noteworthy positive correlation observed between let-7d-5p and the pro-inflammatory cytokine IL-1β (Sondergaard et al. 2013; Tan et al. 2014). Considering that AD, MS, and epilepsy are neural diseases, let-7d-5p may participate in their pathogenesis through shared pathways.

Other microRNAs, such as miR-106b-5p, -15a-5p, -194-5p, and -130a-3p, are involved in inflammation, cell proliferation, and apoptosis in cancers (Cimmino et al. 2005; Hager et al. 2011; Liu et al. 2014; Roccaro et al. 2009; Zhang et al. 2014). In drug-resistant epilepsy patients, miR-194-5p, -4446-3p, -301a-3p, -342-5p, and -30b-5p were remarkedly decreased, targeting inflammation and apoptosis-related genes (Wang et al. 2015a, b). miR-301a-3p plays a role in the inflammatory response through the NF-κB signaling pathway in cancer (Lu et al. 2011). miR-23a, upregulated in various experimental epilepsy profiling studies, plays a role in regulating apoptosis, inflammation, and differentiation-associated transcription factors (Song et al. 2011).

In a pilocarpine-induced epileptic rat model, the decreased expression of miR-322-5p was found to coincide with elevated levels of pro-inflammatory cytokines, increased expression of NF-κB, and decreased GABA levels. Conversely, the introduction of exogenous miR-322-5p resulted in a reduction in inflammation, increased GABA levels, and decreased neuronal apoptosis, suggesting its potential as a novel antiseizure medication (Zhou et al. 2022).

miR-10a expression was found to be increased in vitro, which correlated with decreased PI3K, Akt, and mTOR levels, and increased TNF-α, IL-6, and IL-1β levels. Modulating miR-10a levels affected cytokine secretion and the PI3K/Akt/mTOR pathway, suggesting its potential as a therapeutic target for epilepsy (Lu et al. 2023).

In another study, the effects of miR-136 on TLE were examined, along with the underlying mechanisms involved. TLE rats were treated with miR-136 agomir and assessed for seizure activity, histopathological changes, apoptosis rate, and inflammatory factors. miR-136 overexpression reduced seizures, improved tissue damage, and inhibited inflammation. It also downregulated Wnt/β-catenin pathway-related proteins, offering neuroprotective effects in TLE rats (Cui and Zhang 2022).

The role of miR-106b-5p in epilepsy was explored through investigations utilizing a mouse model and HT22 hippocampal cells. Inhibition of miR-106b-5p was found to facilitate microglia polarization, diminish inflammation, and provide neuronal protection. The RGMa-Rac1-JNK/p38-MAPK signaling axis was identified as being involved in these processes. Targeting miR-106b-5p or its downstream factors could potentially offer therapeutic approaches for the prevention and treatment of epilepsy. (Yu et al. 2022).

Dysregulated lncRNA GAS5, miR-219, and the CaMKIIγ/NMDAR pathway were studied in epilepsy. GAS5 upregulation and miR-219 downregulation were observed. GAS5 knockdown promoted cell proliferation, suppressed inflammation, and apoptosis. GAS5 was found to inhibit miR-219 through EZH2, and the introduction of miR-219 mimics resulted in enhanced cell proliferation, suppressed inflammation, and reduced apoptosis, with modulation by CaMKIIγ. In summary, GAS5 exerted an influence on the inflammatory response and cell apoptosis in epilepsy (Zhao et al. 2022).

The involvement of lncRNA PVT1 in epilepsy has revealed its role in promoting neuroinflammation by modulating the miR-488-3p/FOXD3/SCN2A axis (Wen et al. 2023).

**Fig. 1:**
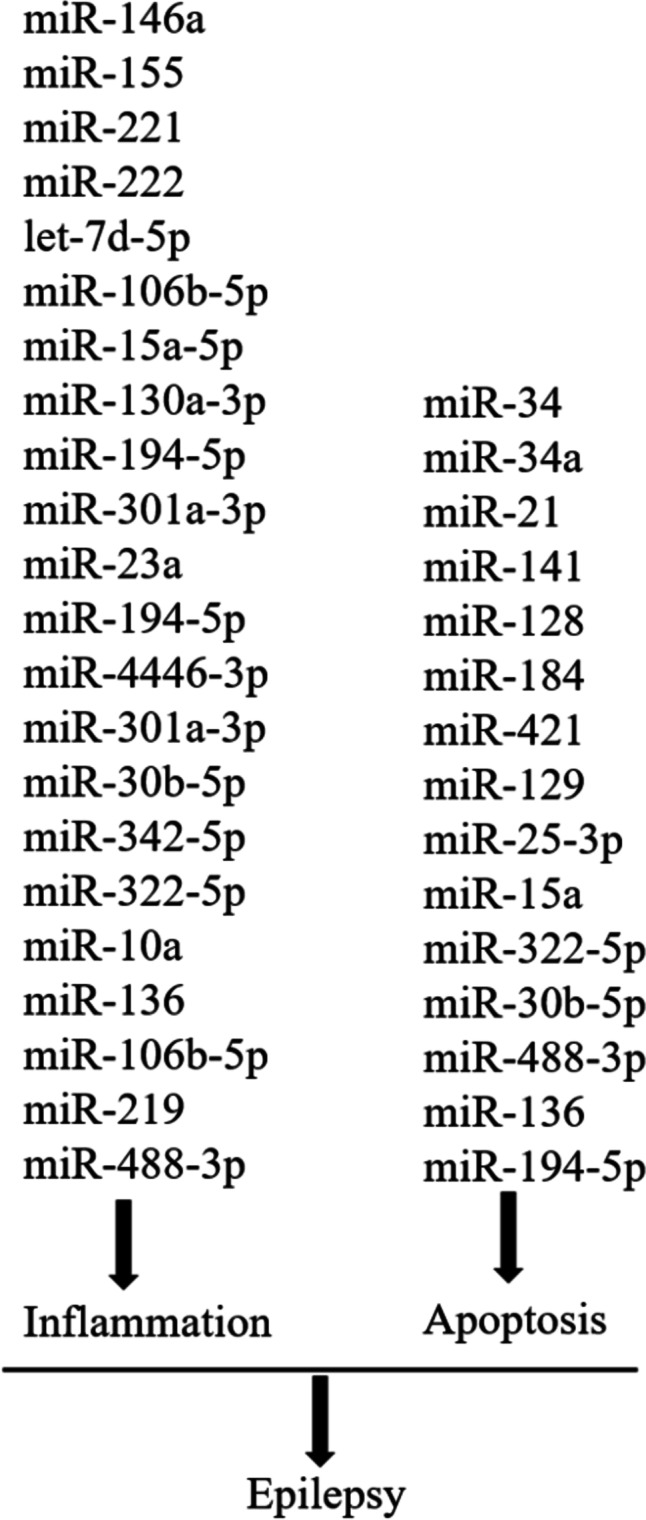
Related miRNAs of inflammation and apoptosis in epilepsy

Manipulating these dysregulated miRNAs **(**Table 2 and Fig. 1) associated with neuroinflammation in epilepsy animal models holds promise for developing innovative therapeutic approaches and investigating their potential as biomarkers in the future.

**Table 2:** The involvement of miRNAs in the inflammatory processes associated with epilepsy

miRNA	Role in Inflammatory Pathways	Study
miR-146a	Negative regulator of TLR signaling Modulates IL-1β-induced inflammatory response by targeting IRAK-1, IRAK-2, TRAF-6, IL-6, COX-2	(Aronica et al. 2010; Cui et al. 2010; Hou et al. 2009; Hu et al. 2011; Iyer et al. 2012; Omran et al. 2012; Saba et al. 2014; Sheedy and O'Neill 2008; Vezzani et al. 2008)
miR-155	Increased expression in hippocampal tissue of children with MTLE linked to increased TNF-α and ICAM-1 levels	(Ashhab et al. 2013)
miR-221 miR-222	Targets ICAM1, involved in inflammation and immune cell interactions Potential role in modulating ICAM1 expression	(Dietrich 2002; Kan et al. 2012; Ueda et al. 2009)
let-7d-5p	Dysregulated in AD and MS, positive correlation with IL-1β in MS	(Sondergaard et al. 2013; Tan et al. 2014)
miR-106b-5p miR-15a-5p miR-130a-3p miR-194-5p	Involved in inflammation, apoptosis, and cell proliferation in cancers	(Cimmino et al. 2005; Hager et al. 2011; Liu et al. 2014; Roccaro et al. 2009; Zhang et al. 2014)
miR-301a-3p	Plays a role in the inflammatory response through the NF-κB signaling pathway	(Lu et al. 2011)
miR-23a	Upregulated in various experimental epilepsy profiling studies, role in regulating apoptosis, inflammation, and differentiation-associated transcription factors	(Song et al. 2011)
miR-194-5p miR-4446-3p miR-301a-3p miR-30b-5p miR-342-5p	Decreased in drug-resistant epilepsy patients, targeting inflammation and apoptosis-related genes	(Wang et al. 2015a, b)
miR-322-5p	Decreased expression, reduced inflammation Increased pro-inflammatory cytokines Increased NF-κB expression Reduced GABA levels	(Zhou et al. 2022)
miR-10a	Increased expression, modulated cytokine secretion Decreased PI3K, Akt, and mTOR levels Increased TNF-α, IL-1β, and IL-6 levels	(Lu et al. 2023)
miR-136	Reduced seizures Improved tissue damage Inhibited inflammation Downregulated Wnt/β-catenin pathway-related proteins	(Cui and Zhang 2022)
miR-106b-5p	Inhibiting miR-106b-5p promoted microglia polarization, reduced inflammation, and protected neurons. Involvement of the RGMa-Rac1-JNK/p38-MAPK signaling axis. Potential therapeutic target for epilepsy	(Yu et al. 2022)
miR-219	GAS5 knockdown promoted cell proliferation, suppressed inflammation, and apoptosis. GAS5 inhibited miR-219 via EZH2. miR-219 mimics enhanced cell proliferation, inhibited inflammation, and apoptosis, modulated by CaMKIIγ	(Zhao et al. 2022)
miR-488-3p	Promote neuroinflammation by LncRNA PVT1 via regulating miR-488-3p/FOXD3/SCN2A axis	(Wen et al. 2023)

Recurrent epileptic seizures can lead to neuronal apoptosis and alterations in synaptic connectivity, contributing to the recurrence of epilepsy. Studies have demonstrated the association between miR-34 and apoptosis, showing that it reduces MAP3K9 levels (Tivnan et al. 2011). After a seizure, the body upregulates pro-apoptotic miRNAs and downregulates anti-apoptotic miRNAs, promoting cell apoptosis and influencing epilepsy development.

Conversely, miR-21 inhibits cell apoptosis and its expression increases in the hippocampus after a seizure, potentially promoting cell apoptosis by reducing the inhibitory effect on neurotrophin-3. Elevated miR-21 levels have been associated with increased caspase-3 protein and apoptotic cell numbers, suggesting its involvement in epilepsy through the activation of pro-apoptotic genes (Peng et al. 2013; Risbud et al. 2011; Wang et al. 2017).

During seizure-induced neuronal death or apoptosis, there is an upregulation of miR-34a, which is another pro-apoptotic miRNA and can be activated by the p53 protein. Targeting miR-34a has shown neuroprotective effects and counteracted the increase in activated caspase-3 protein (Hu et al. 2012). Furthermore, miR-141 and miR-128 are elevated in epilepsy and contribute to nerve cell apoptosis through diverse mechanisms that involve the expression of pro-apoptotic proteins (Chen et al. 2019; Liu et al. 2019). In contrast, some anti-apoptotic miRNAs, such as miR-184, -421, -129, -25-3p, and -15a, have been implicated in epilepsy (R. Li et al. 2020a, b; McKiernan et al. 2012; Wen et al. 2018; Wu et al. 2018). These miRNAs exhibit neuroprotective effects by regulating apoptosis-related genes and pathways. Specifically, miR-184 has anti-apoptotic effects and regulates the Numblike gene (McKiernan et al. 2012). miR-421 downregulation is associated with increased apoptosis and autophagy in hippocampal neurons through the TLR/MYD88 pathway (Wen et al. 2018). Reduced miR-129 expression leads to enhanced proliferation and apoptosis of hippocampal neurons by affecting the c-Fos gene and MAPK signaling pathway (Wu et al. 2018). Downregulation of miR-25-3p is associated with oxidative stress and apoptosis, while downregulated miR-15a contributes to miR-30b-5p increased apoptosis in TLE tissues (R. Li et al. 2020a, b).

In another study, exogenous miR-322-5p administration was observed to reduce neuronal cell apoptosis following SE, along with decreased expression of TLR4, NF-κB, and TRAF6. The results suggest that miR-322-5p is able to inhibit apoptosis may be attributed to its modulation of the TLR4/TRAF6/NF-κB pathway (Zhou et al. 2022).

The regulatory mechanism of the miR-30b-5p/GRIN2A axis in epilepsy was investigated. Restoration of hippocampal neuron proliferation and reduction in apoptosis were observed through the overexpression of miR-30b-5p in vivo. miR-30b-5p targeted GRIN2A to exert its protective effects in EP (Zheng et al. 2023). The involvement of lncRNA PVT1 in epilepsy has revealed its role in promoting apoptosis in neuronal cells by modulating the miR-488-3p/FOXD3/SCN2A axis. (Wen et al. 2023).

The involvement of miR-136 in preventing neuronal cell apoptosis has been identified. Assessment of apoptosis in hippocampal tissue revealed a notably higher rate of apoptosis and elevated expression of c-Caspase-3 in the epilepsy group than the control group, along with reduced expression of the Bcl-2 protein. However, introducing an overexpression of miR-136 led to a remarkable decrease in the apoptosis rate and the expression of c-Caspase-3 in hippocampal tissue, while increasing Bcl-2 protein expression. In hippocampal tissue, the miR-136 + LiCl group demonstrated a notably elevated apoptosis rate and increased expression of c-Caspase-3 compared to the miR-136 group. Additionally, there was a significant decrease in the expression of Bcl-2 protein in the miR-136 + LiCl group compared to the miR-136 group (Cui and Zhang 2022).

The protective effects of glycyrrhizic acid against TLE in young rats have been observed, with its mechanism of action involving the regulation of neuronal ferroptosis through the miR-194-5p/PTGS2 axis. In young rats experiencing TLE, the reduction in prostaglandin-endoperoxide synthase 2 (PTGS2) provided partial alleviation of the heightened iron content and lipid peroxidation in the hippocampus. However, this down-regulation of PTGS2 further intensified neuronal apoptosis and injury, which were initially triggered by the decreased expression of miR-194-5p (Yi et al. 2023).

Collectively, these findings highlight the involvement of various miRNAs (Table 3andFig. 1) in epilepsy pathogenesis and neuroprotection through the regulation of apoptosis. Modulating these differentially expressed miRNAs may offer potential therapeutic approaches for the treatment of epilepsy. Further research is warranted to explore the precise mechanisms and clinical implications of miRNA-mediated apoptosis in epilepsy.

**Table 3:** The involvement of miRNAs in the regulation of apoptosis in epilepsy

miRNA	Role in apoptosis	Study
miR-34 miR-34a	Reduces MAP3K9 levels Upregulated during seizure-induced neuronal death or apoptosis	(Hu et al. 2012; Tivnan et al. 2011)
miR-21	Inhibits cell apoptosis Increased expression after a seizure	(Peng et al. 2013; Risbud et al. 2011; Wang et al. 2017)
miR-141 miR-128	Upregulated in epilepsy Promotes nerve cell apoptosis	(Chen et al. 2019; Liu et al. 2019)
miR-184	Anti-apoptotic effects Regulates the Numblike gene	(McKiernan et al. 2012)
miR-421	Downregulation associated with increased apoptosis and autophagy in hippocampal neurons	(Wen et al. 2018)
miR-129	Reduced expression enhances proliferation and apoptosis of hippocampal neurons	(Wu et al. 2018)
miR-25-3p	Downregulation associated with oxidative stress and apoptosis	(R. Li et al. 2020a, b)
miR-15a	Downregulated in TLE tissues	(R. Li et al. 2020a, b)
miR-322-5p	Reduces neuronal cell apoptosis, downregulates TLR4, NF-κB, and TRAF6 expression	(Zhou et al. 2022)
miR-30b-5p	Restores hippocampal neuron proliferation, reduces apoptosis, targets GRIN2A for protective effects	(Zheng et al. 2023)
miR-488-3p	Regulates PVT1/FOXD3/SCN2A axis, promotes neuronal cell apoptosis in epilepsy	(Wen et al. 2023)
miR-136	Inhibits neuronal cell apoptosis, reduces c-Caspase-3 expression, increases Bcl-2 protein expression	(Cui and Zhang 2022)
miR-194-5p	Regulates PTGS2, protects against TLE, reverses increased hippocampal iron content and lipid peroxidation, but intensifies neuronal apoptosis and injury	(Yi et al. 2023)

## miRNA-based therapy potential in epilepsy

Clinicians can accurately diagnose epilepsy by considering patient history and clinical manifestations. Effective biomarkers play a crucial role in epilepsy diagnosis, classification, and targeted therapy development. Genetic biomarkers such as 5-hydroxy tryptamine (5-HT) receptor gene, sodium channel voltage-gated type I-alpha (SCN1A) gene, the gamma-aminobutyric acid (GABA) receptor gene, inwardly rectifying potassium channel (Kir4.1) gene, and aquaporin-4 (AQP4), along with inflammatory biomarkers including IL-6, TNF-α, and IL-2, can aid in the diagnosis of epilepsy (Symonds et al. 2017). Nevertheless, the use of these biomarkers is constrained due to inconsistent findings and a lack of diagnostic specificity. miRNAs hold great potential as molecular biomarkers for the diagnosis and prognosis of epilepsy, particularly in cases of mTLE. They offer a less invasive option, as blood sampling is sufficient for research purposes. miRNAs exhibit remarkable stability, even under conditions that usually degrade other RNAs, thanks to their interactions with proteins and lipoprotein complexes (Panina et al. 2023; Pitkanen et al. 2016; Vickers and Remaley 2012; Yakovleva et al. 2022). As the understanding of miRNA roles in the pathogenesis of epilepsy deepens, the concept of miRNA-targeted interventions to prevent or delay the onset of epilepsy holds great value. By targeting a single miRNA, multiple cellular processes can be simultaneously regulated, making it a promising intervention strategy following epileptogenic injury. Numerous preclinical studies have showcased the therapeutic potential of miRNAs in treating acute or chronic epilepsy, leading to the initiation of clinical translation. Thus far, miRNA-based therapies have demonstrated favorable tolerability and have shown therapeutic effects in preclinical investigations.

Overexpression of miR-494 repressed RIPK1, leading to inhibition of the NF-κB signaling pathway, enhanced cell proliferation, and reduced apoptosis in hippocampal neurons, ultimately attenuating neuronal injury and epilepsy development (Qi et al. 2020). Through intrahippocampal injection of a specific Agomir in a mouse model of pilocarpine-induced epilepsy, miR-137 was observed to be overexpressed. As a result, the mice experienced an extended latency period of recurrent seizures and a reduction in the severity of epilepsy (W. Wang et al. 2018a, b). miR-219 supplementation suppressed seizures in experimental epilepsy models by modulating the CaMKII/NMDA receptor pathway, suggesting it as a potential therapeutic strategy (Zheng et al. 2016). miR-204 overexpression exhibited anti-epileptogenic effects by downregulating TrkB and modulating the downstream ERK1/2-CREB signaling pathway (Xiang et al. 2015). Silencing miR-134 significantly reduced loss of CA3 pyramidal neurons and abnormal mossy fiber sprouting, demonstrating its therapeutic potential in experimental epileptic seizures (Gao et al. 2019). Ube3am − /p + mice exhibit normal levels of miR-134 and its corresponding targets. However, when an antimiR oligonucleotide inhibitor (Ant-134) specifically targeting miR-134 is intracerebroventricularly injected, it leads to a decrease in the severity of audiogenic seizures and improved behavior in Angelman syndrome (AS) mice. Notably, Ant-134 enhances locomotor activity and exploration as observed in the open-field test. Silencing miR-134 in Angelman patient-derived neurons upregulates its targets, suggesting the therapeutic potential of silencing miR-134 and other microRNAs as a treatment approach for clinically relevant phenotypes in AS (Campbell et al. 2022). By decreasing the levels of miRNA-134 through intracerebroventricular injection of an antagomir, the onset and severity of kainic acid-induced seizures were delayed, and neuronal death in the hippocampus was reduced. These findings suggest that targeting microRNA-134 could be a potential therapeutic approach for managing seizures in children (Campbell et al. 2021). The suppression of miR-129–2-3p in rats exhibited a remarkable capability to reduce seizures, underscoring its potential as a target for the prevention and treatment of refractory epilepsy. The miR-129–2-3p/GABRA1 pathway shows promise as a valuable avenue for future investigations in this field (Wang et al. 2021). Silencing miR-135a in an experimental TLE model reduced seizure activity by regulating Mef2 proteins, key regulators of excitatory synapse density (Vangoor et al. 2019). In mouse models, miR-146a modulation improved epilepsy onset and hippocampal damage by regulating inflammatory factors, showing promise for therapeutic interventions (Tao et al. 2017; X. Wang et al. 2018a, b). Silencing miR-132 inhibited aberrant dendritic spine formation and chronic spontaneous seizures in a lithium-pilocarpine-induced epileptic mouse model, potentially through the miR-132/p250GAP/Cdc42 pathway (Yuan et al. 2016).

The conventional method for experimental overexpression of miRNAs involves using synthetic miRNA mimics, but this approach has limitations such as pathway saturation, off-target effects, and toxicity when delivered to the brain. Gene therapy vectors provide a potential solution by enabling controlled and specific delivery of miRNAs to targeted brain cells. In contrast, the application of antisense oligonucleotide (ASO) antimiRs has become a prominent strategy in reducing miRNA expression. ASO therapies offer lasting gene targeting with infrequent dosing and are progressing towards clinical use. Additional strategies include miRNA-directed short hairpin RNAs, RNA sponges, tough decoy molecules, and target site blockers, each offering unique advantages for inhibiting endogenous miRNA activity. While it is possible to directly administer small molecules into the brain, larger antisense constructs require the use of viral vectors to achieve efficient delivery to cells (Morris et al. 2021). The unique pharmacokinetic (PK) and pharmacodynamic (PD) properties of ASOs have a significant influence on their effectiveness and optimal dosage as therapeutic drugs. In order to enhance the therapeutic potential of antimiR molecules, modifications can be introduced to their molecular structure. ASOs with unmodified sugar structures and phosphodiester (PO) bonds are susceptible to degradation by endo- and exonucleases, which significantly limits their effectiveness. Unprotected ASOs can be rapidly degraded by serum exonucleases within 30 min and even more quickly by intracellular exo- and endonucleases. To overcome this limitation, chemical modifications are incorporated into ASOs to improve their stability. These modifications usually target the 2'-carbon of the sugar ring or PO bond (Eder et al. 1991; Khorkova and Wahlestedt 2017; Lima et al. 2018). ASOs face challenges in crossing the blood–brain barrier (BBB) (Khorkova and Wahlestedt 2017) with only a fraction of oligonucleotides administered intravenously, typically less than 1%, is able to reach the brain (Cossum et al. 1993). To overcome this, alternative approaches are utilized. One strategy involves conjugating oligonucleotides to transport vectors, enabling receptor-mediated endocytosis and transport across the BBB. Cell-penetrating peptides (CPPs) have also shown promise in facilitating cellular uptake of ASOs. Another option is the use of nanoparticle formulations based on poly (lactic-co-glycolic acid) (PLGA), which enhance cellular uptake. Direct delivery techniques, such as intracerebroventricular (ICV) or intrathecal (IT) injection, provide high ASO concentrations in the brain but are invasive. Viral vectors offer cell-type specificity and can deliver larger antisense molecules. Intranasal delivery shows potential for less invasive administration. Furthermore, seizure-induced BBB permeability allows timed systemic ASO delivery (Morris et al. 2021). The clinical trial of miravirsen, the first miRNA-targeting ASO, demonstrated the feasibility of miRNA therapies in humans. Ongoing clinical trials are investigating miRNA therapeutics for CNS and non-CNS diseases, including ALS and kidney disease. In preclinical studies of epilepsy, miR-134-5p has been extensively targeted using ASOs, resulting in a reduction of seizures in various animal models. Other miRNAs, such as miR-135a, -324-5p, -137, -10a-5p, -21a-5p, and -142a-5p, have also shown promise in suppressing seizures in different epilepsy models. Various modifications to ASOs have been employed, including DNA/LNA mixmer, phosphorothioate (PS) linkages, 2'-MOE, and 2'-OMe. Certain ASOs have been modified with a 3'-cholesterol tag to improve cellular uptake, although this alteration can impact solubility. Overall, these studies highlight the potential of miRNA-targeting ASOs as a therapeutic approach for epilepsy and other neurological disorders, with ongoing clinical trials shedding light on their efficacy and safety (Eder et al. 1991; Khorkova and Wahlestedt 2017; Lima et al. 2018).

In conclusion, miRNAs hold great promise as a target for ASO-based therapies in epilepsy and potentially other intricate brain disorders. Further research and development efforts are required to address various challenges and optimize the therapeutic potential of miRNA-targeting approaches.

## miRNAs as Epilepsy Biomarkers

Discovering a molecular biomarker capable of distinguishing epilepsy from both healthy subjects and other neurological conditions would enable the initiation of earlier, more precise diagnosis, and appropriate treatment. miRNAs are one of the potential biomarkers for tracking pathological changes and hold promise as therapeutic targets within the epileptic brain. Regarding potential non-invasive biomarkers for adult TLE and mTLE, noteworthy candidates include miR-142-5p, miR-145-3p, miR-153, miR-199a-3p, and miR-339-5p. Notably, alterations in these miRNAs were also observed within temporal lobe and hippocampal tissue, enhancing their potential relevance in the context of epilepsy. Similarly, miR-142-5p and miR-339-5p showed upregulation, while miR-145-3p exhibited downregulation in the hippocampal tissue of adults with mTLE compared to controls. Furthermore, a downregulation of miR-153 was noted in the temporal cortex tissue of individuals with mTLE compared to controls (Martinez and Peplow 2023). The potential biomarkers identified in cerebrospinal fluid (CSF), namely miR-19b-3p, miR-21-5p, and miR-451a, have shown prior up-regulation after status epilepticus (SE) in brain profiling studies conducted in both rat models (Bot et al. 2013; Gorter et al. 2014; Jimenez-Mateos et al. 2011; Kretschmann et al. 2015; Liu et al. 2010; Peng et al. 2013; Risbud et al. 2011; Roncon et al. 2015; Wang et al. 2015a, b; Wang et al. 2015a, b). Notably, substantial elevation of miR-21-5p has been observed in hippocampal tissues from children with TLE (Peng et al. 2013) and epileptic adults (Roncon et al. 2015) in comparison to control subjects. This altered miR-21-5p expression is thought to counteract edema and mediate anti-apoptotic effects (Buller et al. 2010). Moreover, the biological processes and pathways significantly associated with the validated targets of these miRNAs closely mirror established processes in TLE and SE, encompassing tissue morphogenesis, remodeling, cell differentiation, p53 and apoptosis signaling, as well as Toll-like receptor pathways. Importantly, an overlap exists between the targets of the identified CSF miRNAs and those of miRNAs that have undergone functional validation impacting brain excitability and seizures. While this insight lends mechanistic support to their biomarker potential, it's crucial to acknowledge that miRNA-target predictions are shaped by validation predominantly carried out within cancer and cell biology fields (Godard and van Eyll 2015), with the potential presence of other sources of bias. Interestingly, limited alignment was observed with miRNAs found to be differentially expressed in experimental SE models or serum samples from epilepsy patients, except for miR-21-5p, which exhibited elevated levels in rat plasma a week after experimental status epilepticus (Gorter et al. 2014; Liu et al. 2010; Roncon et al. 2015; Wang et al. 2015a, b; Wang et al. 2015a, b). These findings correspond with observations that underscoring the specificity of miRNA biomarkers to the analyzed biofluid.

Plasma miRNAs were sequenced in mTLE and focal cortical dysplasia (FCD) samples, revealing downregulated miR-134 in mTLE plasma, indicating its potential as a non-invasive MTLE biomarker (Avansini et al. 2017). mTLE patients with hippocampal sclerosis (mTLE-HS) exhibited overexpression of miR-145, miR-181c, miR-199a, and miR-1183 in blood (Antonio et al. 2019). Serum miR-328-3p emerged as a significant MTLE-HS diagnostic biomarker with high AUC values against Engel I controls. miR-654-3p demonstrated predictive strength for surgical prognosis in MTLE-HS patients (Ioriatti et al. 2020). miR-106b-5p was pinpointed as a key diagnostic candidate for epilepsy using Illumina HiSeq2000 sequencing, yielding high sensitivity and specificity (Wang et al. 2015a, b). Up-regulated miR-106b, miR-146a, and miR-301a were detected to be alongside downregulated miR-194-5p in epilepsy patient serum via PCR, with miR-106b and miR-146a correlating with epilepsy severity (An et al. 2016). Their combined detection enhanced sensitivity and specificity for epilepsy prediction. For refractory TLE patients, miR-129–2-3p displayed increased expression in temporal cortex and plasma, with higher epilepsy frequency linked to elevated levels and poor prognosis (Y. Sun et al. 2016a, b). Conversely, plasma miR-145-5p expression decreased significantly in refractory TLE patients, positively associated with age of onset and epilepsy frequency (Shen et al. 2019). It has been observed miR-30a, miR-378, miR-106b, and miR-15a upregulation in serum of epilepsy patients compared to inter-seizure levels, with miR-30a positively tied to seizure frequency (J. Sun et al. 2016a, b). miR-4521 showed upregulation in brain tissue and serum of refractory epilepsy patients, potentially serving as a diagnostic biomarker for refractory focal cortical dysplasia (FCD) (Wang et al. 2016). Elevated miR-323a-5p expression in cortex and plasma of FCD patients with refractory epilepsy suggested potential for monitoring treatment responses (Che et al. 2017). Serum miR-146a and miR-155 levels were elevated in genetic generalized epilepsy patients, and combining miR-132, miR-146a, and miR-155 levels distinguished genetic generalized epilepsy patients from controls with high accuracy (Martins-Ferreira et al. 2020). Certain circulating miRNAs also linked to drug-resistant epilepsy. It has been identified miR-301a-3p as a pivotal biomarker for drug-resistant epilepsy diagnosis, with downregulated expression indicative of drug resistance (Wang et al. 2015a, b). The increased serum miR-134 and miR-146a levels in drug-resistant epilepsy patients, suggesting a higher risk of drug resistance (Leontariti et al. 2020). Ongoing assessment of new epilepsy biomarkers is underway. Expression changes in circulating miRNAs confirmed in epilepsy patients underscore their connection with epilepsy (Antonio et al. 2019; Brennan et al. 2020). To bolster the potential of miRNAs as epilepsy biomarkers, further studies with more cases and consistent miRNA detection methods are needed.

Additional research is also necessary for epilepsy. Exploring the use of mouse, rat, and non-human primate models of epilepsy should also be taken into account. These animal models offer the potential to validate miRNA observations in human patients and to assess the impact of targeting specific miRNAs on both disease advancement and behavioral outcomes.

## Concluding Remarks

miRNAs play a critical role as regulators of gene expression in the progression of epilepsy. Differential miRNA expression enables identification of molecular and cellular alterations in epilepsy patients, highlighting possible targets for therapeutic intervention. However, there is a need to consolidate findings from various studies to establish miRNAs as consistent biomarkers for epilepsy diagnosis. Manipulating pathological genes and disrupting other disease mechanisms hold therapeutic potential. Consequently, the development of effective miRNA therapeutics holds significant promise for advancing epilepsy treatment strategies. Although multiple miRNAs show promise as therapeutic targets for epilepsy, there are still several challenges to be addressed for their clinical application. Studies have primarily focused on single models or species, necessitating validation in diverse etiologies and larger animals. Understanding the mechanisms underlying miRNA-targeted therapy is crucial, yet limited to a small number of studies with scarce in vivo verification. Extensive evaluation of the safety of oligonucleotides targeting brain miRNAs is required through comprehensive preclinical investigations.

## Data Availability

The datasets used and/or analyzed during the current study are available from the corresponding author on reasonable request.
